
JOURNAL INFORMATION
==============================
NLM Title Abbreviation: Biospektrum (Heidelb)
Journal ID: Biospektrum (Heidelb)
NLM Journal ID: 9615685

Biospektrum

ISSN: 0947-0867
EISSN: 1868-6249

ARTICLE INFORMATION
==============================
PMCID: PMC8111366
PMID: 33994673
DOI: 10.1007/s12268-021-1576-6
Article ID: 1576
Article version: 1
Subjects: Wissenschaft

SARS-CoV-PLpro-Inhibitoren als mögliche Breitspektrum-Virostatika

Hammerschmidt Stefan Josef
Müller Patrick
Schirmeister Tanja schirmei@uni-mainz.de

grid.5802.f 0000 0001 1941 7111 Institut für Pharmazeutische und Biomedizinische Wissenschaften (IPBW), Johannes Gutenberg-Universität Mainz, Staudinger Weg 5, D-55128 Mainz, Deutschland
Electronic publication date: 2021 May 11
Print publication date: 2021
Volume: 27
Issue: 3
First page: 254
Last page: 256
Copyright: © Die Autoren 2021
License: Dieser Artikel wird unter der Creative Commons Namensnennung 4.0 International Lizenz veröffentlicht, welche die Nutzung, Vervielfältigung, Bearbeitung, Verbreitung und Wiedergabe in jeglichem Medium und Format erlaubt, sofern Sie den/die ursprünglichen Autor(en) und die Quelle ordnungsgemäß nennen, einen Link zur Creative Commons Lizenz beifügen und angeben, ob Änderungen vorgenommen wurden. Die in diesem Artikel enthaltenen Bilder und sonstiges Drittmaterial unterliegen ebenfalls der genannten Creative Commons Lizenz, sofern sich aus der Abbildungslegende nichts anderes ergibt. Sofern das betreffende Material nicht unter der genannten Creative Commons Lizenz steht und die betreffende Handlung nicht nach gesetzlichen Vorschriften erlaubt ist, ist für die oben aufgeführten Weiterverwendungen des Materials die Einwilligung des jeweiligen Rechteinhabers einzuholen. Weitere Details zur Lizenz entnehmen Sie bitte der Lizenzinformation auf http://creativecommons.org/licenses/by/4.0/deed.de.
License URL: https://creativecommons.org/licenses/by/4.0/

issue-copyright-statement: © Springer-Verlag GmbH Deutschland, ein Teil von Springer Nature 2021

==============================
The SARS-CoV-encoded papain-like cysteine protease (PLpro) plays crucial roles in viral replication and maturation processes. It is required to cleave the precursor polyproteins into functional proteins. Thus, it is considered to be a promising target for developing specific drugs. For rational optimization of hit compounds, information about the structure-activity relationship (SAR) is fundamental. Herein, we characterize isoindolines as a new class of PLpro inhibitors.

▲ **Abb. 1:** Replikationszyklus des SARS-CoV-2. **A**, Vereinfachte Darstellung des Replikationszyklus. Nach Bindung des S-Proteins an ACE2 gelangt das Virion in die Zelle und setzt seine RNA frei. Die RdRp vervielfältigt die RNA, die dann am rauen ER in das Präkursor-pp übersetzt wird. Durch PL$^{pro}$- und M$^{pro}$-katalysierte Spaltung werden die Virusproteine freigesetzt. Die zusammengesetzten Virionen werden aus der Zelle entlassen. **B**, SARS-CoV-2-Präkursor-pp und die Restriktionsstellen, an denen durch PL$^{pro}$ (orange) oder M$^{pro}$ (blau) geschnitten wird.

■ Die SARS-Coronavirus(CoV)-2-Pandemie tobt auf der ganzen Welt und noch ist kein Ende in Sicht. Zurzeit sind keine spezifischen Arzneistoffe zur Behandlung infizierter Patienten zugelassen. Während die meisten Staaten auf flächendeckende Impfungen setzen, die sich ausschließlich auf das Spike(S)-Protein fokussieren, werden nach und nach mehrere „Escape-Varianten" mit Mutationen in diesem S-Protein selektiert. Um dieser Resistenzentwicklung der Viren zu begegnen, ist es von größter Bedeutung, weiterhin an den Grundlagen einer gezielten Arzneimitteltherapie zu forschen. Die hier vorgestellte Arbeit widmet sich der Entwicklung von *small molecules* als Inhibitoren einer für die Virusreplikation essenziellen Protease [1]. Bei Proteasen liegt es nahe, zunächst peptidische Substratanaloga als Hemmstoffe zu entwickeln. Diese verfügen meist jedoch nur über eine geringe Membrangängigkeit, unterliegen raschem Metabolismus und weisen somit reduzierte orale Bioverfügbarkeit auf. Daher liegt der Fokus auf der Entwicklung peptidomimetischer oder im Idealfall nicht peptidischer Strukturen.

## Replikationszyklus der Coronaviren

Viren sind darauf angewiesen, von Wirtszellen repliziert zu werden (**Abb. 1A**). Dazu müssen sie ihre Erbinformation in diese übertragen. Nach Aktivierung durch die Proteasen *transmembrane serine protease type II* (TMPRSS2), Furin und Trypsin kann SARS-CoV-2 an seinen Rezeptor, *angiotensin converting enzyme 2* (ACE2), binden und mittels endosomaler Aufnahme in die Wirtszelle gelangen. Im Lysosom wird nach Aktivierung durch Cathepsin L eine Membranfusion mit dem Virus induziert, was in der Freisetzung von Virusproteinen und der viralen RNA resultiert (**Abb. 1A**). Die viruseigene RNA-abhängige RNA-Polymerase (RdRp) vervielfältigt die RNA. Diese RNA-Kopien werden durch Ribosomen am endoplasmatischen Retikulum (ER) in mehrere Präkursor-Polyproteine (pp) übersetzt. Damit funktionelle Proteine aus den Polyproteinen freigesetzt werden, bedarf es zahlreicher enzymatischer

Spaltungen durch die viruseigenen Cystein-Proteasen *papain-like protease* ($PL^{pro}$) und *main protease* ($M^{pro}$). $PL^{pro}$ schneidet an drei Stellen, während $M^{pro}$ an elf Restriktionsseiten aktiv wird (**Abb. 1B**). Die freigesetzten Proteine können nun ihre jeweiligen Funktionen übernehmen sowie zu neuen Virionen zusammengesetzt werden. Die reifen Virionen werden wieder in den Extrazellularraum entlassen, wo ein neuer Replikationszyklus beginnt.

## Mögliche Ansätze zur Arzneistoffentwicklung

Anhand des Replikationszyklus des SARS-CoV (**Abb. 1A**) lassen sich Zielstrukturen (*targets*) zur Arzneistoffentwicklung ableiten. Ziel hierbei ist es, virusspezifische Prozesse zu stören, um möglichst wenige physiologische Prozesse zu beeinflussen. So soll ein vertretbares Nebenwirkungsprofil erzielt werden. Als neue Ansätze kommen daher Hemmstoffe des Viruseintritts in die Zelle, Polymerase- und Protease-Inhibitoren in Frage:

- ***Entry*-Inhibitoren** verhindern den Eintritt des Virus in die Wirtszelle. Denkbar sind Inhibitoren der TMPRSS2, Furin und Caspase sowie Liganden des Rezeptors ACE2, damit das Virion nicht mehr mit seinem S-Protein binden kann.
- **Polymerase-Inhibitoren:** Die RdRp vervielfältigt die virale Erbinformation und ist virusspezifisch, weshalb hier eine hohe Selektivität möglich ist.
- **Virus-Protease-Inhibitoren:** Die Proteasen $PL^{pro}$ und $M^{pro}$ sind ebenfalls virusspezifisch und sind daher attraktive Zielstrukturen [2].

Die $PL^{pro}$ ist Teil des nicht strukturgebenden Proteins 3 (nsp3) und schneidet zwischen nsp1/nsp2, nsp2/nsp3 und nsp3/nsp4 (**Abb. 1B**). Substrate werden vornehmlich an einem „LXGG↓XX“-Motiv gespalten. Neben dieser Aufgabe übernimmt die $PL^{pro}$ auch weitere für den Replikationszyklus essenzielle Funktionen. Mit ihrer Deubiquitinase-Aktivität moduliert $PL^{pro}$ außerdem die Stabilität von Proteinen und ist an der Regulierung der Immunantwort der Wirtszelle beteiligt [3].

## Strukturähnlichkeit von SARS-CoV und SARS-CoV-2 $PL^{pro}$

Schon bei der ersten SARS-CoV-Pandemie 2002/2003 hat unsere Gruppe an Inhibitoren der $PL^{pro}$ gearbeitet. Um an diese Forschung anzuknüpfen, musste zunächst untersucht werden, inwieweit sich die Proteasen beider SARS-CoV-Stämme ähneln. Ein *alignment* ergab, dass beide Proteasen nahe verwandt sind (*sequence identity* 82 %, *sequence similarity* 90 %), was sich mit einer *root mean square deviation* (RMSD) von 1,8 Å auch in der 3D-Faltung beider Proteasen widerspiegelt.

## Struktur-Wirkungs-Beziehungen von Benzamid- und Entwicklung von Isoindolin-Inhibitoren

Ausgangspunkt unserer SAR-Studie war der Benzamid-Inhibitor (**Abb. 2A**, 1, [4]). Zunächst konnte gezeigt werden, dass die Veränderungen der Benzamidstruktur durch Invertierung der Amidbindung zu Benzanilid-Derivaten oder durch Substitution des Phenylrings zum vollständigen Verlust der inhibitorischen Aktivität führen. Im Weiteren wurde das optimale Substitutionsmuster des Benzamids unter Einbeziehung sowohl sterischer als auch elektronischer Effekte untersucht, wobei sich das 1-Chlorbenzamidderivat (**Abb. 2A**, 2) als einer der potentesten Vertreter dieser Strukturklasse herauskristallisierte. Essenziell sind ebenfalls Konfiguration und Substitution des Chiralitätszentrums: Es werden die (*R*)-Konfiguration und ein 1-Naphthylrest bevorzugt.

Mit der Isoindolin-Verbindung (**Abb. 2A**, 3) konnte ein neues Leitstrukturmotiv für potente $PL^{pro}$-Inhibitoren entwickelt werden. Hauptmerkmale sind zum einen die basische Aminofunktion (berechneter p*K*a ca. 8,5) und die Rigidisierung durch Zyklisierung des Inhibitors. Somit wird der Entropieverlust bei Bindung an das Enzym reduziert. Untermauert wird diese Annahme durch das Fehlen der Aktivität beim azyklischen Analogon. Wie bei den Benzamiden sind die *(R)*-Konfiguration und der 1-Naphthylrest wichtige Strukturmerkmale. Im Gegensatz dazu führt die Einführung der 4-Methylgruppe (analog der 1-Methylgruppe am Benzamidgerüst) oder anderer Substituenten am Isoindolinring zu einer Verringerung der Aktivität.

## Molekulare Dockingstudien

Mithilfe von computergestützten Studien konnte der Bindemodus der Benzamide als auch der Isoindoline postuliert werden

Hier steht eine Anzeige.

Springer

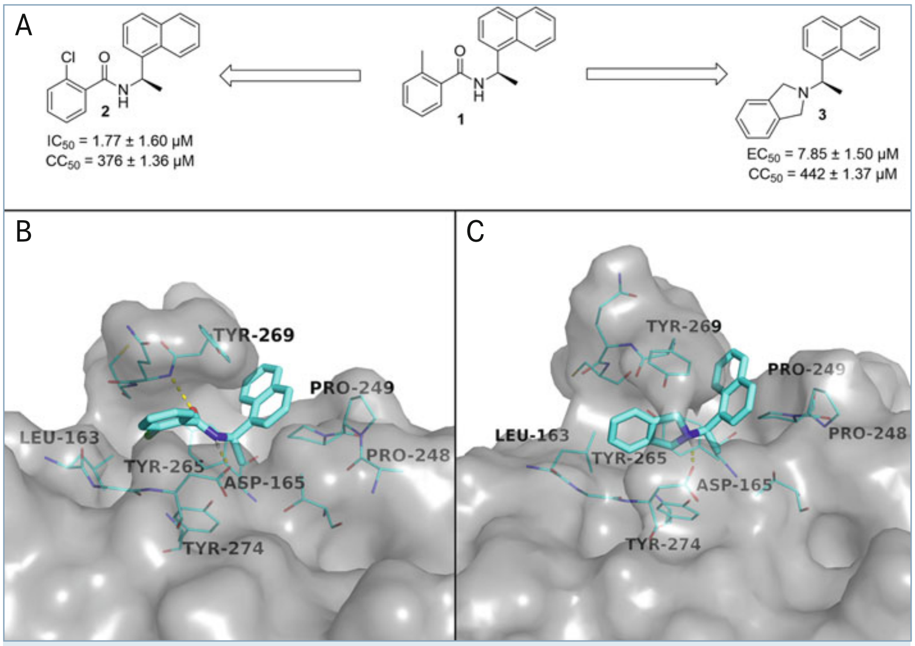

▲ **Abb. 2:** Ansätze zur Optimierung der Hemmstoffe und postulierte Bindemodi. **A**, Ausgehend von **1** wurden die Benzamid- und Isoindolinderivate **2** und **3** entwickelt. **B** und **C**, Berechnete Bindemodi von **2** (B, PDB-ID 3E9S) und **3** (C, PDB-ID 4OW0) im Komplex mit SARS-CoV-$PL^{pro}$. Docking-Abbildungen wurden mit PyMOL generiert.

(**Abb. 2B**, **C**). Dafür wurden zwei Kristallstrukturen der SARS-CoV-$PL^{pro}$ für geladene bzw. ungeladene Liganden als Grundlage verwendet [4–6].

Wie bei Inhibitor 1 adressieren die Naphthylreste beider Inhibitorklassen die hydrophobe S4-Bindetasche, gebildet von Pro248, Pro249 und Tyr269. Das Benzamid- und das Isoindolinstrukturelement finden in der S3-Bindtasche zwischen Leu163, Tyr265 und Tyr274 Platz. Die (*R*)-Konfiguration wird bevorzugt, da Tyr265 aufgrund sterischer Hinderung eine Bindung der *(S)*-konfigurierten Methylgruppe erschwert. Zusätzlich lässt sich eine für die inhibitorische Wirkung essenzielle ionische bzw. polare Wechselwirkung der positiven Aminogruppe der Isoindoline bzw. der Amidgruppe der Benzamide mit Asp165 beobachten.

## Übertragung der SARS-CoV-Daten auf SARS-CoV-2

Um experimentell zu untersuchen, ob die mit SARS-CoV-$PL^{pro}$ erhaltenen Testergebnisse auf die homologe Protease des SARS-CoV-2 übertragen werden können, wurde diese rekombinant exprimiert und isoliert. Wie aufgrund der strukturellen Verwandtschaft beider Enzyme zu erwarten und zu erhoffen, liegen die Aktivitäten bei beiden Proteasen im gleichen Größenbereich. Zusätzlich wurden Daten zur antiviralen Aktivität an SARS-CoV-2 infizierten Vero-6-Zellkulturen und zur Toxizität erhoben. Die Aktivitäten liegen im niedrigen mikromolaren Bereich und mit Selektivitätsindices (SI) von 50–200 treten erst bei deutlich höheren Konzentrationen toxische Effekte auf.

## Ausblick

Durch unsere Forschungsarbeit konnten nicht peptidische, antiviral wirksame Inhibitoren der $PL^{pro}$ von SARS-CoV und SARS-CoV-2 und deren essenzielle Wechselwirkungen mit den Zielenzymen identifiziert werden. Mit Inhibitor 3 wurde für die neue Hemmstoffklasse der Isoindoline ein Vertreter gefunden, der durch die gute Wirksamkeit gegen die $PL^{pro}$ beider Virusvarianten, die antivirale Aktivität in Zellassays und die niedrige Zytotoxizität einen vielversprechenden Startpunkt für die weitere Entwicklung zu potenten $PL^{pro}$-Inhibitoren mit möglicherweise Breitspektrumwirkung darstellt. ■


Funding note

Open Access funding enabled and organized by Projekt DEAL.

Stefan Josef Hammerschmidt 2011–2015 Pharmaziestudium, Universität Mainz. 2016 Approbation als Apotheker. Seit 2018 Promotion im Bereich der Pharmazeutischen/Medizinischen Chemie am Institut für Pharmazeutische und Biomedizinische Wissenschaften (IPBW), Mainz.

Patrick Müller 2013–2019 Studium der Biomedizinischen Chemie an der Universität Mainz. Seit 2020 Promotion im Bereich der Pharmazeutischen/Medizinischen Chemie am Institut für Pharmazeutische und Biomedizinische Wissenschaften (IPBW), Mainz.

Tanja Schirmeister 1988 Approbation als Apothekerin. 1989–1999 Promotion und anschließend Habilitation im Fach Pharmazeutische/Medizinische Chemie. 2000 Hochschuldozentin (C2) am Pharmazeutischen Institut der Universität Freiburg. 2000–2011 C3-Professur für Pharmazeutische/Medizinische Chemie, Universität Würzburg. Seit 2011 Universitätsprofessorin (W3) für Pharmazeutische/Medizinische Chemie, Universität Mainz.


Literatur

[1] Welker A Kersten C Müller C Structure-activity relationships of benzamides and isoindolines designed as SARS-CoV protease inhibitors effective against SARS-CoV-2 ChemMedChem 2021 16 340 354 10.1002/cmdc.202000548 32930481 PMC7894572
[2] Amendola G, Ettari R, Previti S et al. (2021) Lead discovery of SARS-CoV-2 main protease inhibitors through covalent docking-based virtual screening. J Chem Inf Model, 10.1021/acs.jcim.1c0018410.1021/acs.jcim.1c00184 33784094
[3] Shin D Mukherjee R Grewe D Papain-like protease regulates SARS-CoV-2 viral spread and innate immunity Nature 2020 587 657 662 10.1038/s41586-020-2601-5 32726803 PMC7116779
[4] Ratia K Pegan S Takayama J A noncovalent class of papain-like protease/deubiquitinase inhibitors blocks SARS virus replication Proc Natl Acad Sci 2008 105 16119 16124 10.1073/pnas.0805240105 18852458 PMC2571001
[5] Ghosh A Takayama J Rao K Severe acute respiratory syndrome coronavirus papain-like novel protease inhibitors: design, synthesis, protein-ligand X-Ray structure and biological evaluation J Med Chem 2010 53 4968 4979 10.1021/jm1004489 20527968 PMC2918394
[6] Báez-Santos Y Barraza Wilson M X-Ray structural and biological evaluation of a series of potent and highly selective inhibitors of human coronavirus papain-like proteases J Med Chem 2014 57 2393 2412 10.1021/jm401712t 24568342 PMC3983375
